# A Quality Improvement Initiative for Echocardiogram Ordering Patterns in an Academic Hospital

**DOI:** 10.7759/cureus.52717

**Published:** 2024-01-22

**Authors:** Shweta Paulraj, Prashanth Ashok Kumar, Sean Byrnes, Niranjan Ojha, Avneet Singh, Vijay Raj

**Affiliations:** 1 Cardiology, State University of New York Upstate Medical University, Syracuse, USA; 2 Internal Medicine, State University of New York Upstate Medical University, Syracuse, USA; 3 Cardiology/Internal Medicine, State University of New York Upstate Medical University, Syracuse, USA

**Keywords:** high-value care, quality control, echocardiography order, quality improvement, appropriate use criteria

## Abstract

Background

Appropriate Use Criteria (AUC) for echocardiography are a useful tool to deliver quality healthcare. Our quality-based interventional study was designed to assess the trends in appropriate utilization rates for echocardiography in our institution and improve adherence to the AUC criteria for transthoracic echocardiograms (TTE).

Methodology

A prospective, time series analysis was conducted at the Upstate University Hospital for the months of July 2019 and August 2020. A chart analysis was performed on 620 consecutive inpatients who underwent TTE for the month of July 2019. We assessed the trends of the appropriate ordering of TTEs. We then updated our order form incorporating the 42 most common appropriate indications. A post-intervention chart analysis was performed on all inpatient TTEs ordered for the month of August 2020 (n = 410). The appropriateness of the TTE for the entire group was determined based on the true indication per chart review. The primary outcome was the proportion of appropriate and inappropriate TTEs ordered. Secondary outcomes included assessing for concordance between the indication on the order requisition form and by chart review. A p-value <0.05 was considered significant.

Results

Using the 2011 AUC for the entire group, 81% of the pre-intervention TTEs and 79.5% of the post-intervention TTEs were appropriate (p = 0.55). There was a statistically significant reduction in the number of discordant TTE orders before and after the intervention (p < 0.01). In addition, we noted increased appropriateness of TTEs in the concordant group both pre and post-intervention.

Conclusions

Our study demonstrates a significant increase in the concordance between the TTE order sheet and actual indication per chart review with the intervention. This can translate into improved scanning and physician reading quality and time, thereby increasing focus on areas of interest according to the true indication. There was no significant increase in the appropriate TTEs ordered.

## Introduction

Echocardiography is a non-invasive, cost-effective test that can provide a wide array of data for several cardiac and non-cardiac conditions. However, concerns have been raised regarding the overutilization of these tests, especially in scenarios where they are clinically inappropriate [1,2]. This can create an undue burden on echocardiography laboratories while also contributing to unnecessary healthcare spending [3]. Reduction in the orders of inappropriate studies creates an opportunity to minimize healthcare expenditure without a reduction and possibly an improvement in healthcare standards [4].

The American Society of Echocardiography (ASE), the American College of Cardiology Foundation, and the Appropriate Use Criteria Task Force released their latest update of the Appropriate Use Criteria (AUC) for Echocardiography in 2011. The indications for echocardiograms were formulated based on current practice guidelines and common clinical scenarios where echocardiography is frequently considered. A total of 98 indications for transthoracic echocardiograms (TTEs) were scored on a scale of 1 to 9 ranging from inappropriate to appropriate. This is a useful tool to deliver quality healthcare while cutting down unnecessary costs [3]. The objective of our quality-based interventional study was to assess the trends in appropriate utilization rates for echocardiography in our institution and improve adherence to the AUC criteria via an educational quality improvement initiative for TTEs.

## Materials and methods

The study was conducted as a prospective, time series analysis at the Upstate University Hospital tertiary care center for the months of September 2019 and August 2020. The study protocol was submitted to the Institutional Research Board and an exempt status was given.

Data collection was performed using a review of the patients’ electronic records. A chart analysis was performed on 620 consecutive inpatients who underwent TTE from July 1 to July 31, 2019 (n = 620). The initial order requisition form in our hospital only encompassed five appropriate indications with a type-in space provided for any other indications. The charts for these patients were reviewed and the appropriateness of the TTE was determined based on the chart review. The trends of ordering TTEs in our institution per the above review were taken as our pre-intervention analysis.

We then reviewed the indications listed in the AUC from 2011 and condensed them into 42 indications. We updated the order form to incorporate these 42 appropriate indications. A subsequent chart analysis (post-intervention) was performed on all inpatient TTEs ordered over one month (August 2020, n = 410). Patient characteristics, including demographics, the indication for the TTE order per the requisition form and chart review, date of last known TTE, indication number as listed in the AUC, comorbidities including diabetes mellitus, hypertension, hyperlipidemia, history of heart failure, history of arrhythmias, coronary artery disease, cerebrovascular accidents, were documented for each patient.

In both the pre and post-intervention groups, we used the indication derived by a review of the patient’s electronic record (not the indication mentioned on the order requisition form) to determine the true appropriateness of the TTE. The study cohort was then divided into (1) a concordant group (patients in whom the indications listed on the requisition form and by chart review were identical) and (2) a discordant group (patients with varying indications on the requisition form and chart review). If there were multiple potential reasons for a TTE, it was classified under the most appropriate indication.

The exclusion criteria for both the pre and post-intervention arms comprised limited studies that were performed to add missing information, bedside echocardiograms performed by fellows while on-call, and TTEs with insufficient clinical information on the records to assign an indication. In patients who underwent more than one complete TTE during the study period, each study was included independently in the analysis.

The primary outcome was the proportion of appropriate and inappropriate TTEs ordered. Secondary outcomes included assessing for concordance between the indication on the order requisition form and by chart review.

Analysis was performed with MedCalc Version 20.218. Data were expressed as percentages and mean ± standard deviation as appropriate. Descriptive statistics and the chi-square test were used for analyzing data, and a p-value <0.05 was considered to be significant.

## Results

The total number of echocardiograms reviewed during the study was 1,030 (pre-intervention = 620, post-intervention = 410). The mean age of the population studied was 63.1 years in the pre-intervention arm and 62.3 years in the post-intervention arm. The demographic data are summarized in Table 1.

**Table 1:** Univariate analysis of baseline characteristics of our patient population. * = statistically significant.

Variable	Pre-intervention (n = 620)	Post-intervention (n = 410)	Pvalue
Age in years (mean ± SD)	63.1 ± 17	62.3 ± 16.1	
Male	51.94%	58.05%	0.0541
Hypertension	396 (63.81%)	269 (65.61%)	0.5547
Dyslipidemia	296 (47.74%)	193 (47.07%)	0.8331
Diabetes mellitus	188 (30.32%)	130 (31.71%)	0.6366
Coronary artery disease	134 (21.61%)	83 (20.24%)	0.5978
Heart failure	166 (26.77%)	87 (21.22%)	0.0429*
Cerebrovascular accident	117 (18.87%)	53 (12.9%)	0.0115*
Arrhythmia	169 (27.26%)	62 (15.12%)	<0.0001*

**Figure 1:**
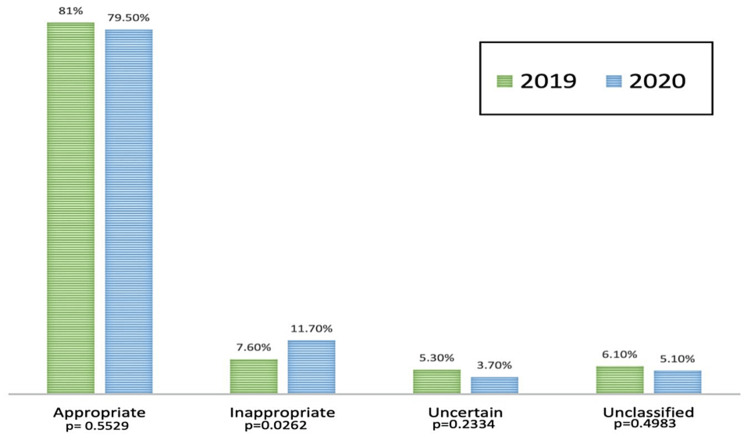
Appropriateness of TTEs in 2019 and 2020. TTE = transthoracic echocardiogram

Using the 2011 AUC for the pre-intervention group, 81% of TTEs were ordered for appropriate indications, 7.6% were for inappropriate indications, 5.3% were for uncertain indications, and 6.1% were in the unclassified category not defined in AUC. In the post-intervention arm, 79.5% of the TTEs were ordered for appropriate indications, which was not significantly different from the pre-intervention arm (p = 0.5529). There was, however, a significant increase in inappropriate TTEs when compared to the prior year (11.7%, p = 0.0262) (Figure 1). The distribution of our patient population across the AUC scores is summarized in Table 2.

**Table 2:** Distribution of appropriate, inappropriate, and uncertain echocardiograms.

	Pre-intervention (%)	Post-intervention (%)	
Score 9	428 (69.03%)	284 (69.27%)	Appropriate
Score 8	55 (8.87%)	35 (8.54%)	
Score 7	19 (3.06%)	7 (1.71%)	
Score 6	11 (1.77%)	7 (1.71%)	Uncertain
Score 5	18 (2.9%)	8 (19.51%)	
Score 4	4 (0.64%)	0 (0)	
Score 3	13 (2.09%)	20 (4.88%)	Inappropriate
Score 2	33 (5.32%)	28 (6.83%)	
Score 1	1 (0.16%)	0 (0)	
Unclassified	38 (6.13%)	21 (5.12%)	
Total	620	410	

**Figure 2:**
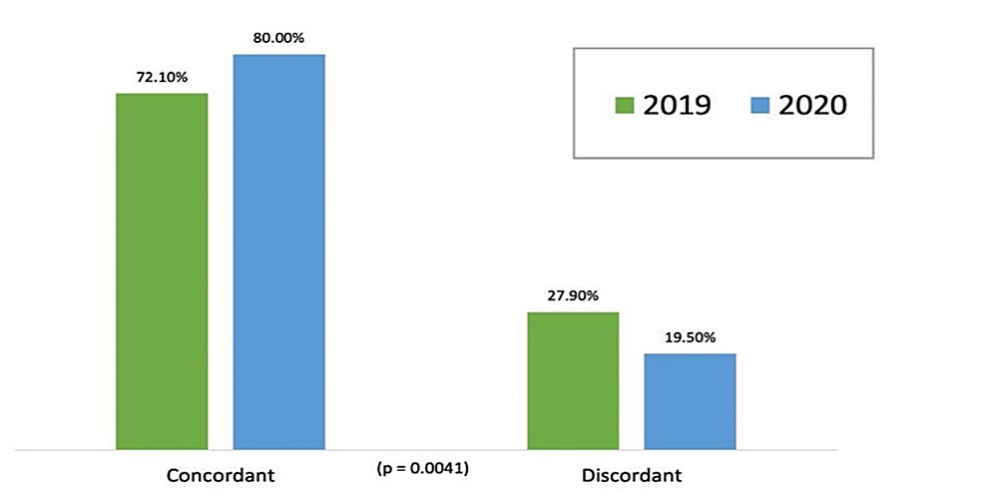
Concordant and discordant TTEs in 2019 and 2020. TTE = transthoracic echocardiogram

We also identified concordant requests. A statistically significant increase in the number of concordant TTE orders was observed after the intervention (p = 0.0041) (Figure 2).

In both the pre and post-intervention arms, we noted a significantly higher number of appropriate echocardiogram orders in the concordant group compared to the discordant group (p < 0.0001). We also noted a significantly higher number of inappropriate and unclassified echocardiograms in the discordant group (Table 3).

**Table 3:** Difference in the level of appropriateness between concordant and discordant groups.

		Appropriate	Inappropriate	Uncertain	Unclassified
Pre-intervention	Concordant	385	23	14	19
	Discordant	117	24	19	19
	P-value	<0.0001	0.0004	0.0002	0.0032
Post-intervention	Concordant	279	29	10	10
	Discordant	47	19	5	11
	P-value	<0.0001	0.0003	0.1889	0.0001

In both the pre and post-intervention arms, the most common indication for which an echocardiogram was ordered was “Symptoms or conditions potentially related to suspected cardiac etiology including but not limited to chest pain, shortness of breath, palpitations, TIA, stroke, or peripheral embolic event” which is an appropriate indication with an AUC score of 9. The most common unclassified indication was for intracranial hemorrhage. Finally, the most common inappropriate indication was “Routine perioperative evaluation of ventricular function with no symptoms or signs of cardiovascular disease” with an AUC score of 2.

## Discussion

In our study, we evaluated the application of the 2011 AUC criteria and the prevalence of appropriate clinical indications in a university-based tertiary care setting.

Our findings showed that approximately 80% of the echocardiograms ordered both pre and post-intervention were appropriate. The prevalence of appropriate echocardiogram orders both within and outside of the United States has been estimated to be similar [5-9].

The most common indication for an echocardiogram at our institution was indication #1 of the AUC “Presence of symptoms or conditions potentially related to suspected cardiac etiology including but not limited to chest pain.” This has similarly been the most common indication in multiple other studies evaluating the use of AUC in their institutions [8,10,11].

The number of inappropriate studies at our institution was estimated to be about 1 in 10 [5-9]. Interestingly, we noted an increase in inappropriate studies despite the intervention. This could be accounted for in the setting of fewer studies performed in the post-intervention arm. On an analysis of the baseline characteristics of the patients in the pre and post-intervention arm, we noted a significantly lower number of patients with heart failure, cerebrovascular accidents, and arrhythmias in the post-intervention arm. The most common inappropriate indication for ordering an echocardiogram was indication #13 (Perioperative evaluation). Our study reflects the data from prior studies showing an inappropriate ordering of echocardiograms as a part of routine perioperative evaluation of ventricular function with no symptoms and signs of heart disease. Multiple studies have reiterated the lack of improved survival or shorter hospital stays with routine preoperative echocardiograms [12-14].

This highlights the need for targeted educational initiatives to minimize the ordering of inappropriate echocardiograms in this area. We suggest a prompt with the ordering of echocardiograms for this particular indication to minimize the frequency of the same.

Prior studies based on the 2007 AUC criteria reported 11%-16% unclassifiable indications for the echocardiogram [15-17]. With the advent of the 2011 AUC criteria, the prevalence of unclassifiable indications has ranged from 0.4% to 4.39% [8,10,11]. Our study showed a higher percentage of unclassified studies, although there was a statistically insignificant decrease of 6.1%-5.1% with the intervention. The decline is likely secondary to an increasing number of indications that can be picked on the requisition form. The most common reason for an unclassified study was intracranial hemorrhage. The proportion of studies for this specific order was 44.07% of all unclassified studies in both arms combined. In a literature review, we identified literature suggestive of transient neurocardiogenic myocardial dysfunction and stunning with prognostic correlation of echocardiographic parameters after an aneurysmal subarachnoid hemorrhage [18-21]. This may be a valuable addition to the current AUC criteria.

An interesting aspect of our study was the identification of an increased number of appropriate studies in the concordant group and an increased number of inappropriate studies in the discordant group. While we noted a significant increase in concordant studies post-intervention, it did not translate into an increase in appropriateness in the post-intervention arm. However, the increase in concordance may translate into focused scanning. The ASE recommends a sufficient time allocation of about 45-60 minutes for the acquisition of images for a complete TTE [22]. An increase in concordant indications can facilitate improved physician reading time by more focus on specific disease pathology targeting the question asked by the clinical team. The increased concordance in our study was likely secondary to an increased number of indications listed in the requisition form, thereby enabling the clinical team to pick a more suitable indication. We did include an option to type in indications not included in the requisition form.

Our intervention did not notice a decrease in inappropriate echocardiograms with the intervention. Additionally, the intervention was performed during the COVID-19 pandemic which may also have had an impact on the echocardiogram ordering patterns. This suggests that merely increasing the availability of indications in the order form without targeted educational initiatives may not have a significant impact on the trends of ordering echocardiograms. Studies have suggested a decline in inappropriate echocardiograms with educational intervention, although the long-term success of the same requires sustained education and feedback [23]. Moreover, ordering prompts for TTEs have only minimally reduced the number of TTEs ordered with decay over time [24].

In summary, adherence to the AUC can help ensure maximum utilization of the best practices. It can serve as a good starting point to ensure quality improvement in echocardiogram utilization [25].

Minimizing discordant ordering of tests will also minimize the focus on irrelevant diagnoses which may also lead to inappropriate management.

Study limitations

This was a cross-sectional study involving a single center with a limited population. In addition, our intervention involved making changes to the echocardiogram order requisition form. No formal education was provided to the ordering physicians regarding the AUC criteria which may have contributed to the lack of a significant increase in appropriateness of echocardiogram orders.

Finally, several of the AUC 2011 criteria are based on a consensus which may represent useful guidelines to evaluate appropriateness but are not the hallmark to assess the clinical relevance of practice in echocardiography [3].

## Conclusions

In our study, 81% of TTEs in the pre-intervention and 79.5% in the post-intervention arm were ordered for appropriate indications. We noted a decrease in the unclassified echocardiograms with the institution of an updated order requisition form. However, we did not see a decline in inappropriate echocardiograms (11.7% post-intervention). There was a significant increase in concordance between indications on the order requisition form and per chart review which may translate to improved scanning times and more focused, clinically relevant physician reads on the echocardiogram. Furthermore, recognition of unclassified clinical scenarios facilitates the identification of areas that would benefit from future research. AUC implementation can facilitate minimizing inappropriate use of healthcare resources and can have a positive impact on clinical outcomes as well as physicians. The initiation of a quality improvement project can help identify the most common causes for inappropriate echocardiograms ordered in an institution and thereby target educational initiatives in that area.
